# Insulin treatment increases myocardial ceramide accumulation and disrupts cardiometabolic function

**DOI:** 10.1186/s12933-015-0316-y

**Published:** 2015-12-18

**Authors:** Aimee E. Hodson, Trevor S. Tippetts, Benjamin T. Bikman

**Affiliations:** Department of Physiology and Developmental Biology, Brigham Young University, 3017 LSB, Provo, UT 84602 USA

**Keywords:** Insulin, Hyperinsulinemia, Mitochondria, Ceramide

## Abstract

**Background:**

States of hyperinsulinemia, particularly insulin resistance and type 2 diabetes mellitus, are becoming remarkably common, with roughly half a billion people likely to suffer from the disorder within the next 15 years. Along with this rise has been an associated increased burden of cardiovascular disease. Considering type 2 diabetics treated with insulin are more likely to suffer from heart complications, we sought to determine the specific effect of insulin on ceramide-dependent cardiometabolic risk factors, including insulin resistance and altered heart mitochondrial physiology.

**Methods:**

H9c2 cardiomyocytes and adult mice were treated with insulin with or without myriocin to inhibit ceramide biosynthesis. Insulin and glucose changes were tracked throughout the study and mitochondrial bioenergetics was determined in permeabilized cardiomyocytes and myocardium.

**Results:**

Herein, we demonstrate that insulin is sufficient to disrupt heart mitochondrial respiration in both isolated cardiomyocytes and whole myocardium, possibly by increasing mitochondrial fission. Further, insulin increases ceramide accrual in a time-dependent manner, which is necessary for insulin-induced alterations in heart mitochondrial respiration and insulin resistance.

**Conclusions:**

Collectively, these observations have two implications. First, they indicate a pathological role of insulin in heart complications stemming from mitochondrial disruption. Second, they identify ceramide as a possible mediator of insulin-related heart disorders.

## Background

We have known for decades that type 2 diabetes mellitus diabetes (T2DM) increases the risk of heart disease [1]. Indeed, the observation is so common that this phenomenon is referred to as “diabetic heart disease”, bringing attention to the fact that cardiovascular complications are the most common cause of mortality in those suffering with T2DM [2, 3]. Considering the increasing incidence of T2DM worldwide [4], and the remarkable number of undiagnosed cases, at least in early stages [5], understanding the nature of the relationship between these two pathologies may prevent heart disease and prolong healthy living among those with T2DM.

Reflective of the prevailing understanding of the etiology of T2DM, a great deal of research efforts have focused on glucose and glycemic control as the causal factors between T2DM and heart disease [6–10]. This focus has elucidated several glucose-related mechanisms, such as the reduction of glucose to sorbitol [11], and especially, the formation of advanced glycation end-products (AGE) and activation of its receptor (RAGE) [12–14]. Moreover, whether a consequence of RAGE activation or a distinct mechanism, hyperglycemia is known to induce inflammation [15]. Similarly, poor glycemic control may disrupt mitochondrial function and increase production of reactive oxygen species [16].

However, while the focus on glucose as a mediating mechanism linking T2DM to cardiovascular complications has yielded valuable insight, it nevertheless ignores what may be at least an equally relevant etiological factor of T2DM etiology—insulin. Pories and Dohm recently posited that excess insulin, not glucose, is the essential factor in T2DM onset [17], a position supported by considerable evidence [18]. T2DM is a progressive spectrum of insulin resistance, with overt T2DM representing a state where insulin secretion, despite being elevated, is no longer sufficient to control blood glucose. As some have recommended a paradigm shift from looking at diabetes as a consequence of hyperinsulinemia rather than hyperglycemia, we are prompted to explore the causal relationship between T2DM and heart disease in a similar light.

Previous reports have observed a role for insulin in the etiology cardiovascular complications [19]. Importantly, insulin therapy, despite adequately controlling blood glucose, has been shown to increase mortality in T2DM [20]. Similar to glucose-induced mechanisms (e.g., AGE formation, etc.), insulin has distinct downstream mediators; one mediator may be the sphingolipid ceramide. Ceramides are increasingly recognized as an injurious mediator of heart pathologies [21–25] and we have recently found that insulin increases ceramide biosynthesis and accrual in skeletal muscle [26, 27]. In light of the evidence suggesting a role for insulin in the etiology of heart complications, the purpose of these experiments was to determine the effect of insulin on heart ceramides, as well as possible ceramide-induced alterations in mitochondrial function.

## Methods

### Cell culture

H9c2 cardiomyocytes were maintained in DMEM +10 % FBS. For differentiation into myotubes, cells were grown to confluency and the medium was replaced with DMEM +10 % horse serum (Invitrogen, Grand Island, NY). Myotubes were used for experiments on day 3 of differentiation. Cells were treated with insulin (50 nM; Actrapid; Novo Nordisk, Plainsboro, NJ) and myriocin (10 µM; Sigma), an inhibitor of serine palmitoyltransferase, at the times indicated.

### Animals

Sixteen-week-old male C57Bl/6 mice were separated into one of four groups (six per group) to receive morning injections of saline (PBS), insulin (daily; 0.75 U/kg/BW; Actrapid; Novo Nordisk, Plainsboro, NJ), myriocin (thrice weekly; 0.3 mg/kg; Sigma) or both for 28 days with free access to water and chow (Harlan 8604) throughout the length of the study. After the 28-d treatment, mice underwent intraperitoneal glucose (G7021; Sigma-Aldrich, St. Louis, MO) and insulin (Actrapid; Novo Nordisk, Plainsboro, NJ) tolerance tests. For both tests, mice were fasted for 6 h and received an injection of either glucose (1 g/kg body wt) or insulin (0.75 U/kg body wt). These are doses that are above the typical rate of insulin treatment in type 2 diabetics (0.5 U/kg) [28]. Plasma glucose (Bayer Contour glucose meter), insulin (ELISA; Crystal Chem Inc.), and adiponectin (Crystal Chem Inc.) levels were determined. Studies were conducted in accordance with the principles and procedures outlined in the National Institutes of Health Guide for the Care and Use of Laboratory Animals and were approved by the IACUC (Institutional Animal Care and Use Committee) at Brigham Young University.

### Lipid isolation analysis

Lipids were extracted and quantified as described previously [29]. Briefly, lipids were isolated with chloroform–methanol (1:2), and after addition of water, the organic phase was collected and dried. After resuspension, lipids were quantified using a shotgun lipidomics technique on a Thermo Scientific LTQ Orbitrap XL mass spectrometer.

### Protein analysis

Cell and tissue proteins were analyzed via western blot as described previously [29].

### Cell and myocardium permeabilization

For cells, H9c2 cardiomyocytes were detached in culture dishes with 0.05 % trypsin–EDTA (Sigma) and growth medium was added to the culture. Contents were transferred to a tube and centrifuged for 10 min at 1000×*g* at RT. After removal of supernatant, cells were lifted in MiR05 [0.5 mM EGTA, 3 mM MgCl_2_, 60 mM K-lactobionate, 20 mM taurine, 10 mM KH_2_PO_4_, 20 mM HEPES, 110 mM sucrose, and g/l BSA (Sigma; A3803) adjusted to pH 7.1] plus 1 mg/ml digitonin and gently rocked at RT for 5 min before centrifugation at 1000×*g* for 5 min. After discarding supernatant, cells were then suspended in 2.2 ml warm MiR05 and transferred to chambers in the O2K (Oroboros Instruments, Innsbruck, Austria). Following respiration protocol (outlined below), cells were removed from the chambers and used for protein quantification. For myocardial mitochondrial respiration, left ventricle was quickly removed from euthanized mice and immediately placed in ice-cold buffer X (60 mM K-MES, 35 mM KCl, 7.23 mM K_2_EGTA, 2.77 mM CaK_2_EGTA, 20 mM imidazole, 20 mM tuarine, 5.7 mM ATP, 15 mM PCr, 6.56 mM MgCl_2_–6H_2_O, pH 7.1) and trimmed of connective tissue. Small fiber bundles were prepared and gently separated along their longitudinal axis under a surgical scope (Olympus, ST) to 1–2 mg. Bundles were then transferred to a tube with chilled buffer X and 50 μg/ml saponin and rocked at 4 °C for 30 min, then washed in buffer Z (105 mM K-MES, 30 mM KCl, 10 mM KH_2_PO_4_, 5 mM MgCl_2_–6H_2_O, 0.5 mg/ml BSA, pH 7.1) at 4 °C for at least 15 min. Samples were then blotted dry and weighed.

### Mitochondrial respiration protocol

High-resolution O_2_ consumption was determined at 37 °C in permeabilized cells and fiber bundles using the Oroboros O2 K Oxygraph with MiR05 respiration buffer. Before addition of sample into respiration chambers, a baseline respiration rate was determined. After addition of sample, the chambers were hyperoxygenated to ~300 nmol/ml. Following this, respiration was determined by all or parts of the following substrate-uncoupler-inhibitor-titration (SUIT) protocol: electron flow through complex I was supported by glutamate  +  malate (10 and 2 mM, respectively) to determine leak oxygen consumption (GM_*L*_). Following stabilization, ADP (2.5 mM) was added to determine oxidative phosphorylation capacity (GM_*D*_). Succinate was added (GMS_*D*_) for complex I  +  II electron flow into the Q-junction. To determine full electron transport system capacity in cells over oxidative phosphorylation, the chemical uncoupler carbonyl cyanide 4-(trifluoromethoxy) phenylhydrazone (FCCP) was added (0.05 μM, followed by 0.025 μM steps until maximal O_2_ flux was reached). Mitochondrial membrane integrity was tested in all experiments by adding cytochrome *c* (not shown; 10 μM). Lastly, residual oxygen consumption was measured by adding antimycin A (2.5 μM) to block complex III action, effectively stopping any electron flow, which provides a baseline rate of respiration.

### Statistics

Data are presented as the mean  ±  SEM. Data were compared by ANOVA with Tukey’s post hoc analysis (Graphpad Prism; La Jolla, CA). Significance was set at *p* < 0.05.

## Results

### Insulin increases cardiomyocyte ceramide accrual, which is necessary for mitochondrial disruption

**Fig. 1:**
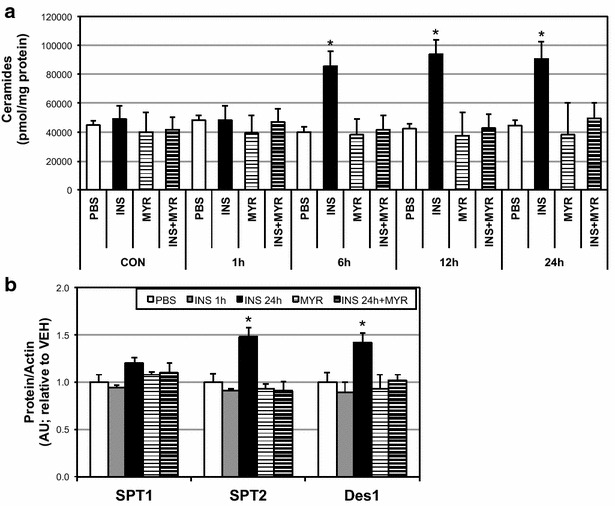
Insulin increases ceramide in cardiomyocytes. H9C2 cardiomyocytes were treated with insulin (INS; 50 nM) with or without myriocin (MYR; 10 μM), an inhibitor of ceramide biosynthesis, for the times indicated (n = 6). Following treatment time, lipids were isolated for analysis of sphingolipids via LCMS (**a**; n = 6) and protein levels of ceramide biosynthetic enzymes determined (**b**; n = 4). *P < 0.05 for INS vs. other treatments

**Fig. 2:**
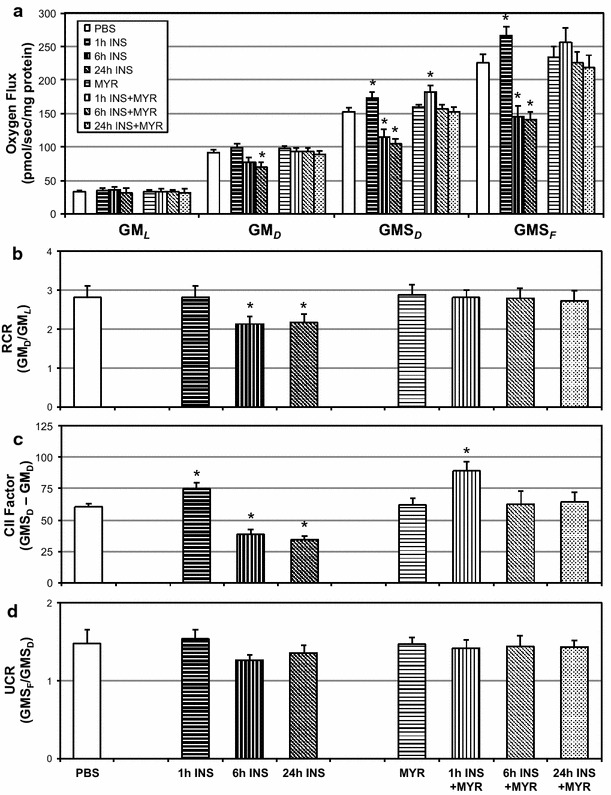
Ceramide inhibition prevents insulin-induced mitochondrial disruption. H9C2 cardiomyocytes were treated with insulin (INS; 50 nM) with or without myriocin (MYR; 10 μM), an inhibitor of ceramide biosynthesis, for the times indicated (n = 6). To measure mitochondrial respiration (**a**), cells were treated with: GM_*L*_, Glutamate (10 mM) + Malate (2 mM); GM_*D*_: + ADP (2.5 mM); GMS_*D*_, + Succinate (10 mM); GMS_*F*_, + FCCP (0.05 μM). Respiratory control ratio (RCR; **b**), Complex II Factor (**c**), and uncoupling control ratio (UCR; **d**) were determined by the analysis indicated. *P < 0.05 for condition vs. control (PBS)

We observed a significant time-dependent increase in ceramide accrual in cardiomyocytes with insulin treatment (Fig. 1a), which was supported with an increase in heart levels of serine palmitoyltransferase 2 (SPT2) and dihydroceramides desaturase 1 (Des1) with insulin treatment (Fig. 1b) at 24 h. Whereas 1 h of insulin had no effect on ceramides, 6 h of treatment roughly doubled ceramide levels, a level maintained, but not significantly increased, at 12 and 24 h. With previous mitochondrial-specific effects of ceramides in mind [23, 25], we next determined whether this ceramide accrual had any effect on cardiomyocyte mitochondrial bioenergetics. Insulin altered mitochondrial respiration in a contrasting and time-dependent manner. At 1 h of insulin treatment, respiration was increased in cardiomyocytes, but significantly decreased at 6 and 24 h (Fig. 2a). Insulin-induced alterations in respiration were also evident in the reduced respiratory control ratio, an overall indication of mitochondrial health [30], at longer time points (Fig. 2b). Moreover, distinct function of complex II-mediated respiration, defined as the CII factor, revealed acutely increased (at 1 h) then decreased (at 6 and 24 h) respiration rates (Fig. 2c). Overall, uncoupling control ratio, calculated as maximal uncoupled respiration by FCCP relative to ADP-stimulated state, was comparable among all conditions (Fig. 2d). Importantly, inhibition of ceramide accrual with myriocin abolished the insulin-induced decrement in respiration (Fig. 2a–c).

**Fig. 3:**
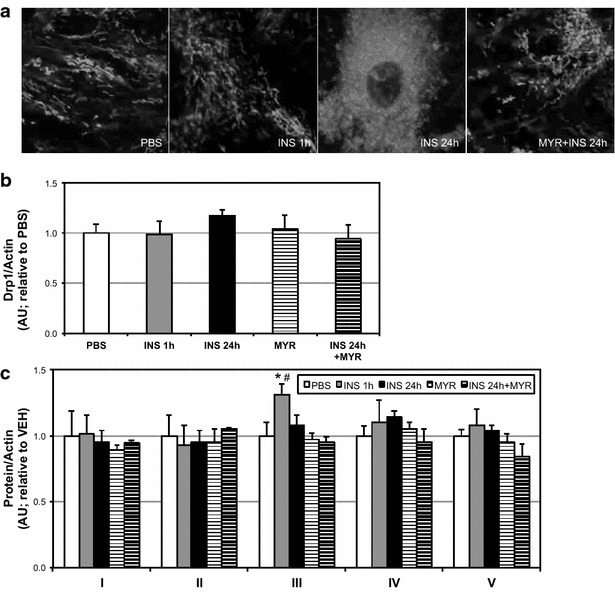
Insulin treatment affects cardiomyocyte mitochondrial physiology. H9C2 cardiomyocytes were treated with insulin (INS; 50 nM) with or without myriocin (MYR; 10 μM), an inhibitor of ceramide biosynthesis, for the times indicated. Following treatment, cells were imaged to determine mitochondrial morphology (**a**; n = 3), and analyzed for Drp1 protein levels (**b**; n = 4), and mitochondrial complex proteins (**c**; n = 4). *P < 0.05 for INS vs. PBS; ^#^P < 0.05 for INS + MYR vs. INS alone

Additionally, we found that insulin altered mitochondrial morphology, appearing to increase mitochondrial fission (Fig. 3a), which was prevented with ceramide inhibition. Drp1 levels were similar among all treatments (Fig. 3b). Further, 1 h of insulin treatment was associated with an increase in mitochondrial complex III levels, but this was lost with 24 h (Fig. 3c).

### Insulin treatment increases body mass and causes hyperinsulinemia and insulin resistance in mice

**Fig. 4:**
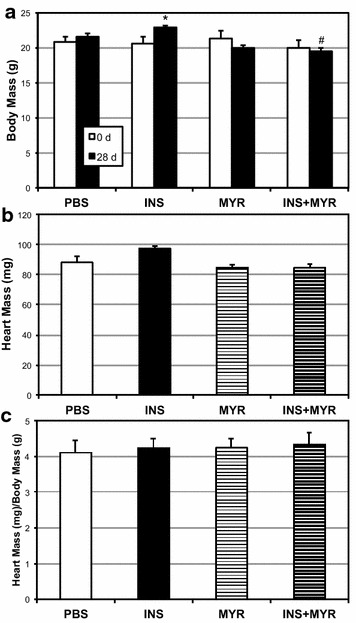
Insulin injections increase body mass, but not heart mass in mice. 16-week-old male mice received injections of PBS (daily), insulin (INS; daily; 0.75 mg/kg), myriocin (MYR, thrice weekly; 3 mg/kg), or INS + MYR for 28 d. Body mass increased in the INS-treated mice only (**a**; n = 6). Heart mass was measured in all mice (**b**, **c**; n = 6). *P < 0.05 for INS vs. PBS; ^#^P < 0.05 for INS + MYR vs. INS alone

**Fig. 5:**
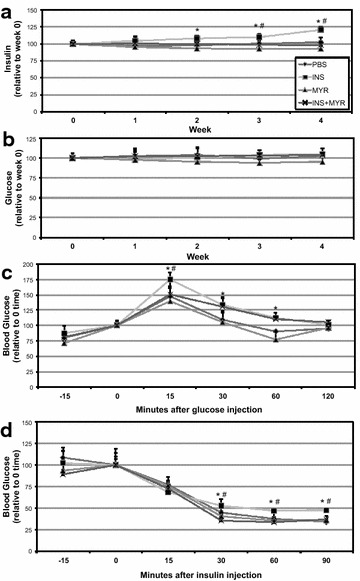
Chronic insulin injections increase blood insulin and induce glucose and insulin intolerance. 16-week-old male mice received injections of PBS (daily), insulin (INS; daily; 0.75 mg/kg), myriocin (MYR, thrice weekly; 3 mg/kg), or INS + MYR. Blood insulin (**a**) and glucose (**b**) was tracked weekly. At the conclusion of the study, IP glucose (**c**) and insulin (**d**) tolerance tests were performed. *P < 0.05 for INS vs. PBS; ^#^P < 0.05 for INS + MYR vs. INS alone

To determine whether an in vivo correlate exists to substantiate our in vitro findings, we injected adult male mice (16 week old) with insulin daily (0.75 mg/kg) for 28 days. At the conclusion of the 28 days treatment period, mice injected with insulin (INS) gained significantly more body mass than PBS-injected mice (Fig. 4a); however, those injected with insulin (daily) and myriocin (thrice weekly; INS + MYR) did not gain such mass. Moreover, those injected with myriocin alone (MYR) weighed less than PBS-injected mice. While heart mass tended to increase with INS injection (Fig. 4b; P = 0.072), the change was not significant, and was even less remarkable when controlled for by body mass (Fig. 4c). INS-injected mice also had higher fasting insulin (Fig. 5a), but not glucose (Fig. 5b) over the course of the treatment. Further, INS treatment caused compromised glucose and insulin tolerance (Fig. 5a, b, respectively), but not with ceramide inhibition.

### Insulin treatment in mice increases muscle ceramides and alters mitochondrial bioenergetics and morphology

**Fig. 6:**
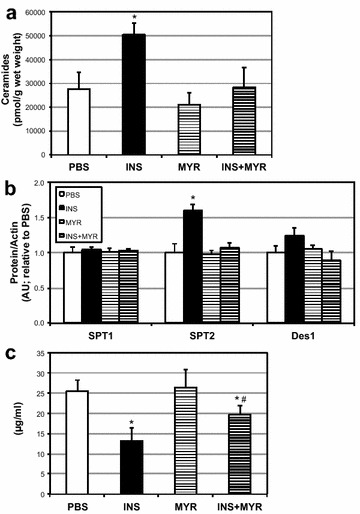
Insulin injection increases heart ceramides. 16-week-old male mice received injections of PBS (daily), insulin (INS; daily; 0.75 mg/kg), myriocin (MYR, thrice weekly; 3 mg/kg), or INS + MYR. INS treatment increased myocardial ceramide accrual (**a**) and SPT2 (b). Serum adiponectin was also measured (**c**). *P < 0.05 for condition vs. PBS. ^#^P < 0.05 for INS + MYR vs. INS alone

**Fig. 7:**
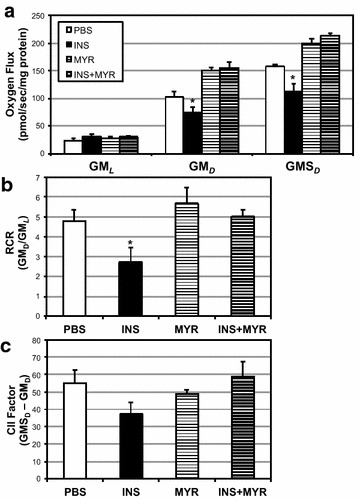
Chronic insulin injections disrupt myocardial mitochondrial function. 16-week-old male mice received injections of PBS (daily), insulin (INS; daily; 0.75 mg/kg), myriocin (MYR, thrice weekly; 3 mg/kg), or INS + MYR. Mitochondrial assessments were determined in permeabilized (saponin, 50 µg/ml) myocardium. To measure mitochondrial respiration (**a**), samples were treated with: GM_*L*_, Glutamate (10 mM) + Malate (2 mM); GM_*D*_, + ADP (2.5 mM); GMS_*D*_, + Succinate (10 mM). Respiratory control ratio (RCR; **b**) and Complex II Factor (**c**) were determined by the analysis indicated. *P < 0.05 for condition vs. PBS

**Fig. 8:**
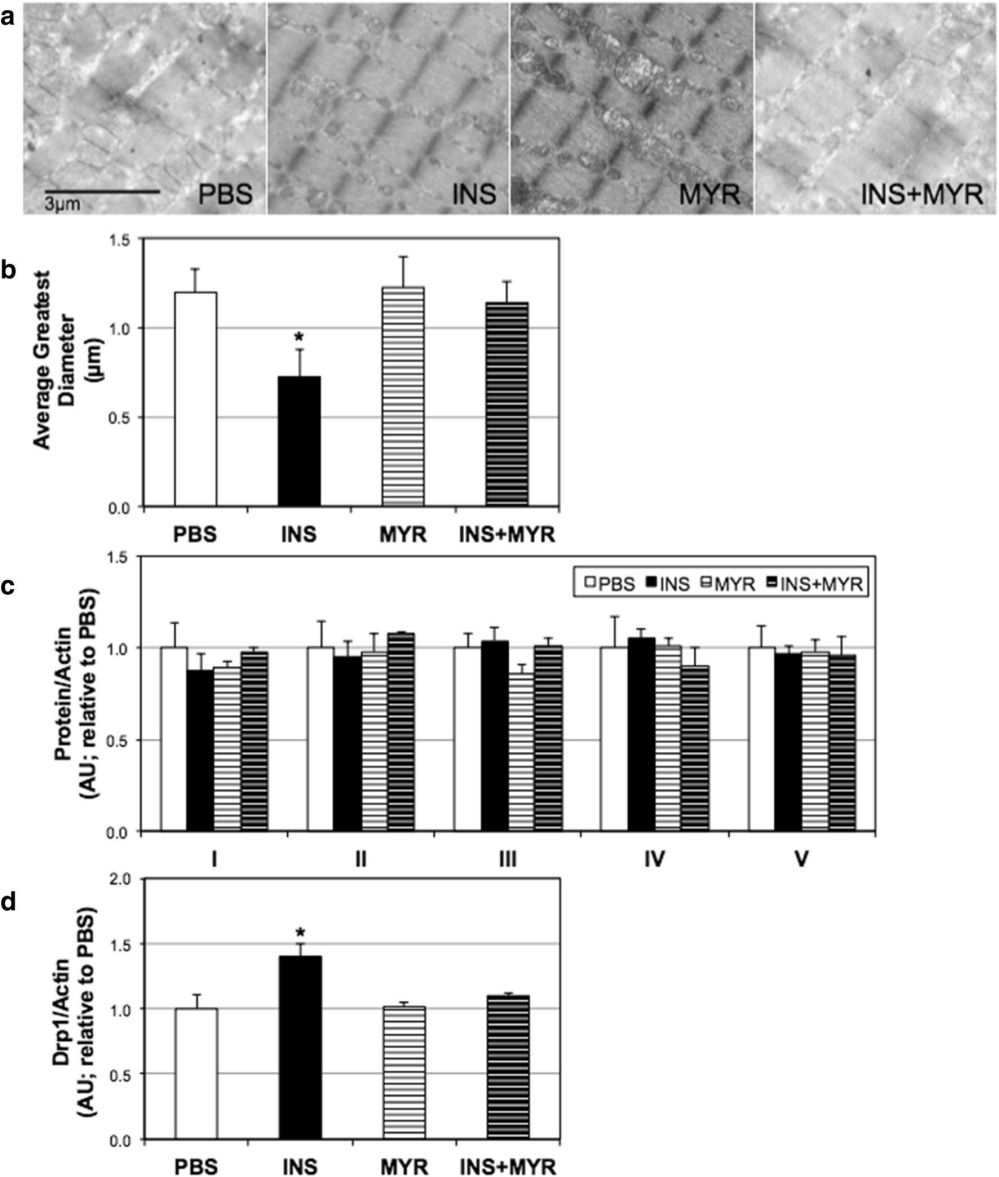
Chronic insulin injections disrupt myocardial mitochondrial function. 16-week-old male mice received injections of PBS (daily), insulin (INS; daily; 0.75 mg/kg), myriocin (MYR, thrice weekly; 3 mg/kg), or INS + MYR. Heart samples were processed for imaging via electron microscopy (**a**) and quantified based on average greatest mitochondrial diameter (**b**; n = 3). A portion of samples was used to probe for mitochondrial complexes (**c**; n = 3) and Drp1 (D; n = 3). *P < 0.05 for condition vs. PBS

28 days of insulin elicited a roughly two-fold increase in myocardial ceramide content (Fig. 6a), though myriocin co-treatment prevented this effect. This effect was supported with an increase in heart SPT2 levels (Fig. 6b). Moreover, blood adiponectin was robustly inhibited with INS and moderately protected with MYR injections (Fig. 6c). Functionally, the increased ceramide accrual had a demonstrable and deleterious effect on myocardial mitochondrial respiration. In particular, overall respiration and RCR was reduced with INS treatment (Fig. 7a, b), though CII factor was not significantly changed (Fig. 7c). Lastly, we found that myocardial mitochondria were smaller with INS treatment compared with all other conditions (Fig. 8a, b), though this was not reflected in any change in levels of mitochondrial complex proteins (Fig. 8c). This effect may be a result of INS-induced increased Drp1 levels in the heart (Fig. 8d).

## Discussion

Type 2 diabetes carries an increased risk of developing a surprising and increasing number of pathologies. Multiples lines of evidence reveal its hand in diseases stemming from cognitive [31], reproductive [32], musculoskeletal [33], and cardiovascular disorders [34]. However, type 2 diabetes is typified by two key characteristics—hyperglycemia and hyperinsulinemia [35, 36]—and while the disease has historically been defined by blood glucose levels, insulin may be a more sensitive and relevant diagnostic [37]. Indeed, a very recent study found that higher insulin exposure in type 2 diabetics is associated with a threefold increase in cardiovascular events [38]. Herein, we demonstrate that chronic insulin injections exert a time-specific and ceramide-dependent effect on cardiometabolic function, including insulin resistance and heart mitochondrial changes.

These studies provide additional insight into the etiology of type 2 diabetes-related heart complications. In particular, these results suggest that insulin is an important pathogenic mediator and highlight the need to regularly measure insulin when evaluating heart disease risk. Our findings of insulin impacting mitochondrial physiology are not new—Parra et al. [39] found that insulin increased mitochondrial respiration. However, while we tended to see an overall dampening effect of insulin on mitochondrial respiration, a notable difference between our studies is the length of time; this previous report used a 3-h incubation, while we used several time points in our in vitro model. Indeed, our data corroborate those of Parra et al. [39] when we analyzed mitochondrial respiration at 1 h, but not at periods over 6 h. Combined with our observations following a 4-week insulin treatment in mice, these data collectively suggest the clinical relevance of prolonged increases in insulin.

In mice, we found that prolonged insulin treatment resulted in reduced glucose and insulin tolerance, suggesting that insulin alone, independent of other variables, is capable of inducing insulin resistance. This observation corroborates evidence from several previous reports in humans and rodents wherein hyperinsulinemia from endogenous (e.g., insulinoma) [40] and exogenous (e.g., injections) [26, 41, 42] sources causes insulin resistance. This insulin-desensitizing effect of prolonged hyperinsulinemia is likely at least partially mediated via ceramide accrual [26]. While it is possible that the insulin-resistant state caused by the insulin treatment in our study exerts some confounding effect on altering heart mitochondrial function independent of insulin-induced heart ceramide accrual, we nonetheless consider this an apparent feature of the prolonged hyperinsulinemia. Nevertheless, insulin resistance per se, in the absence of the often-accompanying hyperinsulinemia, may be the responsible lesion.

In light of the observations by Dohm and Pories [17], who implicate hyperinsulinemia in the etiology of T2DM, we submit an alternative hypothesis as to the origins of diabetic heart disease that should be considered. As opposed to heart disease being a consequence of the potentially harmful milieu associated with T2DM, perhaps heart disease and T2DM are each consequences of one pathology—hyperinsulinemia. Such a theory is supported by multiple reports that implicate insulin alone in the etiology of both heart disease [43–45] and T2DM [46, 47]. Over two decades ago, Haffner et al. [48] wondered about the role of insulin and queried whether heart disease started before diabetes onset, when insulin, but not glucose, is elevated. Their results add to the body of evidence that insulin is an important etiological factor in heart disease. A significant strength of measuring insulin is that it is elevated earlier than glucose in the progression to frank T2DM [46, 47] allowing not only an earlier diagnosis, but also an earlier, and thus more effective, intervention. Collectively, these observations emphasize the need to measure insulin in routine health screenings.

Altogether, these results highlight the pathogenicity of hyperinsulinemia on cardiometabolic function, including insulin resistance and heart mitochondria. These findings are corroborated by recent work by Marciniak et al. [49] who found reduced cardiac mitochondrial function in a mouse model of type 2 diabetes, with concomitant hyperinsulinemia. Interestingly, cardiac mitochondrial function was largely unaffected in the streptozotocin-induced model of type 1 diabetes, which strengthens the insulin-centric paradigm of altered cardiometabolic health with type 2 diabetes. Another finding from Marciniak et al. [49] was that adiponectin was reduced in their model of type 2 diabetes, but not type 1, which is a common finding in humans [35]. Considering the actions of adiponectin signaling on ceramide metabolism [50] and cardiovascular function [51], the reduced adiponectin that accompanies most insulin-resistant conditions may provide additional explanation into the increased heart ceramide accrual and reduced adiponectin we observed in our model of directly induced hyperinsulinemia [52].

The purpose of these studies was to explore the effect of insulin in altering cardiometabolic function, with a focus on two main components: insulin resistance and heart mitochondrial dynamics and physiology. However, while our findings shed light on the role of insulin in cardiometabolic pathologies, they nevertheless fall short of allowing firm conclusions concerning cardiovascular health. Thus, a significant weakness that will need to be addressed in future studies is the lack of analyses to determine a *functional* impairment with the heart in this same context.

## Conclusions

Our data suggest two potential therapeutic strategies for mitigating the heart disease burden associated with states of elevated insulin (e.g., pre-diabetes or T2DM). First, drugs to induce insulin sensitization (e.g., metformin) should take priority over drugs that induce insulin secretion (e.g., sulfonylurea), which is associated with a reduction [53] and increase [54–56] in heart disease risk, respectively. Second, ceramide inhibition may prove to be an effective deterrent to heart disease risk in various conditions, including hyperinsulinemia, as mounting evidence suggests inhibition of ceramide biosynthesis is effective at protecting cardiovascular health [57–59].
